# Genomic, ecological, and morphological approaches to investigating species limits: A case study in modern taxonomy from Tropical Eastern Pacific surgeonfishes

**DOI:** 10.1002/ece3.5029

**Published:** 2019-03-05

**Authors:** William B. Ludt, Moisés A. Bernal, Erica Kenworthy, Eva Salas, Prosanta Chakrabarty

**Affiliations:** ^1^ National Museum of Natural History Smithsonian Institution Washington District of Columbia; ^2^ Department of Biological Sciences 109 Cooke Hall State University of New York at Buffalo Buffalo New York; ^3^ Ichthyology Section, 119 Foster Hall, Museum of Natural Science, Department of Biological Sciences Louisiana State University Baton Rouge Louisiana; ^4^ FISHBIO Santa Cruz California

**Keywords:** Niche modeling, Phylogeography, *Prionurus*, Reef fish, Ultraconserved elements

## Abstract

A wide variety of species are distinguished by slight color variations. However, molecular analyses have repeatedly demonstrated that coloration does not always correspond to distinct evolutionary histories between closely related groups, suggesting that this trait is labile and can be misleading for species identification. In the present study, we analyze the evolutionary history of sister species of *Prionurus* surgeonfishes in the Tropical Eastern Pacific (TEP), which are distinguished by the presence or absence of dark spots on their body. We examined the species limits in this system using comparative specimen‐based approaches, a mitochondrial gene (COI), more than 800 nuclear loci (Ultraconserved Elements), and abiotic niche comparisons. The results indicate there is a complete overlap of meristic counts and morphometric measurements between the two species. Further, we detected multiple individuals with intermediate spotting patterns suggesting that coloration is not diagnostic. Mitochondrial data recovered a single main haplotype shared between the species and all locations resulting in a complete lack of structure (Φ_ST_ = 0). Genomic analyses also suggest low levels of genetic differentiation (*F*_ST_ = 0.013), and no alternatively fixed SNPs were detected between the two phenotypes. Furthermore, niche comparisons could not reject niche equivalency or similarity between the species. These results suggest that these two phenotypes are conspecific and widely distributed in the TEP. Here, we recognize *Prionurus punctatus* Gill 1862 as a junior subjective synonym of *P. laticlavius *(Valenciennes 1846). The underlying causes of phenotypic variation in this species are unknown. However, this system gives insight into general evolutionary dynamics within the TEP.

## 1 INTRODUCTION

Species are the fundamental unit of biology, and as such their proper identification is critical for a variety of disciplines, including phylogenetics, biogeography, population genetics, and conservation (De Queiroz 2005). Traditionally species are diagnosed by one or more morphological differences (either fixed or in combination) between groups of organisms. In groups that generally display vibrant coloration patterns, such as tropical coral reef fishes, many species have been delimited through subtle color differences (Leray et al., 2010; Rocha, 2004; Taylor & Hellberg, 2005). For many reef fishes, color or squamation patterns have been used to identify genetic breaks between major biogeographic provinces (DiBattista et al. 2013; Coleman et al. 2016), and to detect areas with high rates of endemism, such as Hawaii (Randall & Rocha, 2009) and the Marquesas (Gaither et al. 2015). However, a number of studies have shown that differences in color patterns are not always indicative of reduced gene flow (Ramon, Lobel, & Sorenson, 2003; Lin, Sanchez‐Ortiz & Hastings, 2009; Schultz et al. 2007), and can be discordant with patterns of genetic structure (DiBattista et al., 2015; Gaither et al., 2014; Leray et al., 2010). Taken together, these studies indicate that color patterns alone are not well‐suited for defining species limits, but should be used in concert with other measurements to ensure an accurate reflection of evolutionary history.

The tropical Eastern Pacific (TEP) is a marine biogeographic region that spans 29° of latitude from Magdalena Bay, Mexico, to the Gulf of Guayaquil, Ecuador (Robertson & Cramer, 2009). Numerous studies have categorized the TEP into three to five biogeographic provinces based on the distribution records of fishes (Briggs, 1974; Briggs & Bowen, 2012; Hastings, 2000; Robertson & Cramer, 2009; Spalding et al., 2007). This region has been partially isolated from the Indo‐Pacific since the Miocene, and completely separated from the Atlantic since the closure of the Isthmus of Panama. The biodiversity of the TEP pales in comparison to that of its neighboring Central/West Pacific region, and it has consequently been discussed as having “reduced speciation capacity,” particularly in several iconic reef‐fish families (Cowman & Bellwood, 2013). Still, speciation within the TEP is facilitated by the limited connectivity between the offshore islands and the continental coast (Allen & Robertson, 1994). Examples include the high rates of fish endemism of the Galapagos (~17% endemic species), Clipperton atoll (~7% endemic species), Cocos Island (~4%), and the Revillagigedos (~8%; Cortés, 2012; Robertson & Cramer, 2009). Many of these offshore endemics are distinguished by coloration differences from their continental congeners, and for some groups, multiple offshore islands have their own endemic species. For example, in *Holocanthus* angelfishes, *H. clarionensis* and *H. limbaughi *occur on the Revillagigedos and Clipperton Islands, respectively, and diverged from their widespread mainland sister species, *H. passer*, ~1.4 mya (Alva‐Campbell, Floeter, Robertson, Bellwood, & Bernardi, 2010; Tariel, Longo, & Bernardi, 2016). Divergence between oceanic and continental species has been detected at a variety of time scales, suggesting that no single oceanographic event led to the isolation of coastal and oceanic populations, and that limited connectivity between these ecosystems repeatedly promotes speciation (Alva‐Campbell et al., 2010; Craig, Hastings, Pondella, Ross Robertson, & Rosales‐Casián, 2006; Tariel et al., 2016; Wainwright et al., 2018).

Not all speciation in the TEP is between offshore islands and the mainland, as sister species are also distributed latitudinally along the continental coast (Hastings, 2000; Riginos, 2005). In many cases, coastal speciation is observed in fishes with reduced dispersal capabilities, such as those with demersal eggs or short pelagic larval durations (e.g., blennies; Eytan, Hastings, Holland, & Hellberg, 2012; Lin & Hastings, 2011; Miller, Lin, & Hastings, 2016). However, this is not always the case, as fishes with high dispersal potential are hypothesized to have diverged in situ in coastal habitats, such as grunts (Bernal et al. 2017; Bernardi, Alva‐Campbell, Gasparini, & Floeter, 2008; Rocha, Lindeman, Rocha, & Lessios, 2008; Tavera, Acero, Balart, & Bernardi, 2012), wrasses (Wainwright et al., 2018), and *Prionurus *surgeonfishes (Ludt, Rocha, Erdmann, & Chakrabarty, 2015).

**Figure 1:**
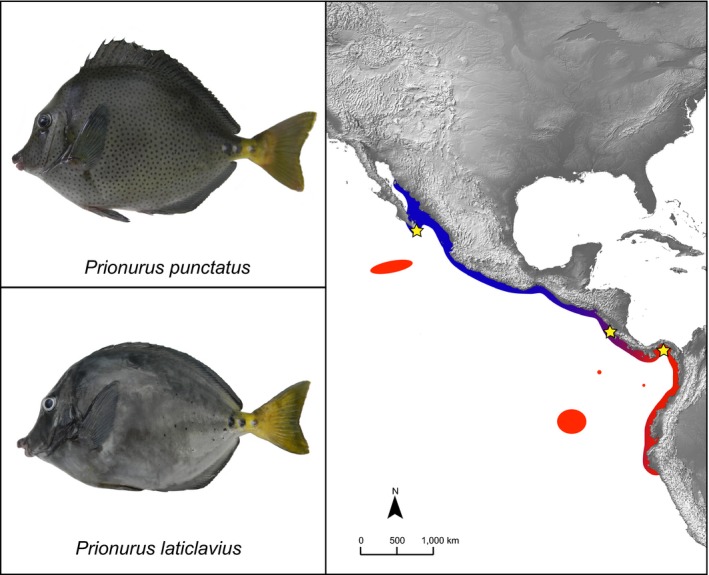
Distribution of two TEP species of surgeonfishes. *Prionurus punctatus *(upper left) is shown in blue, and *P. laticlavius* (lower left) is shown in red. Yellow stars show the sampling locations for this study. The offshore islands are previously only thought to be occupied by *P. laticlavius*. However, two vouchered specimens of *P. punctatus* have been verified from the Revillagegedos

The present study focuses on two species of *Prionurus* surgeonfishes distributed latitudinally throughout the TEP: *P. punctatus *occurs from the Gulf of California to Costa Rica, while *P. laticlavius *extends from Costa Rica to Ecuador, also occupying offshore islands of the TEP (Figure 1; Robertson & Allen, 2015). This pattern of distribution is somewhat unexpected, as surgeonfishes have extremely high dispersal potentials (Doherty, Planes, & Mather, 1995), and several species lack population structure across entire ocean basins (Dibattista, Wilcox, Craig, Rocha, & Bowen, 2011; Eble, Rocha, Craig, & Bowen, 2011; Eble, Toonen, & Bowen, 2009). In fact, while seven surgeonfish species regularly occur in the TEP (Allen & Robertson, 1994), the two species of *Prionurus* are the only surgeonfishes in the region that are not also present in the Indo‐Pacific. Furthermore, these two species are nearly identical phenotypically. In the description of *P. punctatus*, Gill notes that “it widely differs from the previously known [*P. laticlavius*] by its spotted body; in other respects it is most nearly allied to the *Prionurus laticlavius *from the Galapagos Islands” (Gill 1862). The situation is further complicated by a recent phylogenetic analysis of the genus, where a multilocus approach did not recover these two species as reciprocally monophyletic (Ludt et al., 2015). However, that particular study was based on three individuals of *P. punctatus* and two of *P. laticlavius*, and it is possible that the loci did not provide the resolution needed to distinguish shallow divergences (Ludt et al., 2015). Considering their distribution across the continental waters of the TEP, as well as their morphological and phylogenetic similarities, it would be interesting to explore potential differences at the genomic level that could diagnose *P. punctatus *and *P. laticlavius*. This would clarify the status of these species, while providing insight into the patterns of genomic divergence of closely related species in the region.

Here, we expand upon the results of Ludt et al. (2015) by including individuals from several locations across the TEP and by adding genomic analyses between the two species. In addition to genetic data, we gathered traditional morphological and meristic data for both species across their ranges and compared them to original species descriptions and type material. We then examined if ecological factors may be responsible for any divergences between these species in order to assess possible speciation drivers along the coastal TEP.

## 2 MATERIALS AND METHODS

### 2.1 Phenotypic and morphological comparisons

To assess species limits in this system, both molecular and specimen‐based approaches were used. An in‐depth morphological comparison of these two species has never been conducted and could reveal more characters consistent with species diagnoses than just squamation patterns. For this purpose, specimens for *P. punctatus* and *P. laticlavius* were examined from across their distributions for phenotypic and morphological variation. Standard measurements and meristic counts were taken for each specimen following those reported in Randall (2001). This included counting the spines and rays of the dorsal, anal, and pectoral fins, and measuring the body depth, predorsal length, pelvic‐fin and anal‐fin lengths in proportion to standard length. The two species mainly differ in the presence or absence of dark spots covering the body; thus, photographs of all specimens were taken to determine how consistent spotting pattern is as a character across the entire TEP. All measurements were made with digital calipers, and averages were calculated for each species.

### 2.2 Molecular sampling and extraction

To have a better understanding of the genetic divergence between *P. punctatus *and *P. laticlavius* along the mainland TEP, we sampled at three localities: Baja California, Mexico; Guanacaste, Costa Rica; and Las Perlas Islands, Panama. This sampling scheme targets two extreme locations, where only a single species is reported in the literature (Mexico for *P. punctatus*, and Panama for *P. laticlavius*), as well as one location where the two species overlap in their recorded distributions (Guanacaste, Costa Rica). Samples were obtained between 2012 and 2015 using either nets along the shore or pole spears while SCUBA diving. Tissue samples were taken from pectoral fins, gills, or muscle tissue and stored in 95% EtOH. Once in the laboratory, tissue samples were stored in a −80°C freezer prior to sample preparation. When possible, voucher specimens were fixed in formalin and deposited at the Louisiana State University Museum of Natural Science.

Genomic material was extracted from each sample using the Qiagen DNeasy Blood and Tissue extraction kit following manufacturers protocols. Extracts were then quantified using a Qubit 2.0 fluorometer with a dsDNA BR Assay Kit (Life Technologies). Quality of genomic extractions was assessed via gel electrophoresis, with a 1% agarose gel using SYBR Safe DNA gel stain (Invitrogen) and 6x blue/orange loading dye (Promega). All extracts were then kept at −20ºC prior to library preparation and amplification.

### 2.3 Mitochondrial sequencing and analysis

To determine if our increased sampling effort was enough to resolve the relationships of these two species, we amplified all samples for the mitochondrial COI barcoding region. Primers and PCR reactions protocols were identical those described in Ludt et al. (2015) and can be found in the appendix. All samples were purified and sequenced in both forward and reverse directions using the Genomic Sequencing and Analysis Facility at the University of Texas at Austin. Sequencing was performed on an Applied Biosystems 3730 sequencer. All sequences were edited and aligned using Geneious 6.0.5 (Biomatters), and all alignments were checked manually. Haplotype networks were created using the TCS networks option in PopART (Clement, Posada, & Crandall, 2000). Summary statistics (haplotype and nucleotide diversities, Φ_ST_), and Fu's *F* statistic (Fu, 1997) were calculated using Arlequin 3.5 (Excoffier, Laval, & Schneider, 2005). An AMOVA was conducted to test for population structuring between the two species, as well as between sampling localities, using 50,000 permutations in Arlequin. These summary statistics were calculated for both species and for all sampling locations.

### 2.4 Genomic library preparation, sequencing, and analysis

For each sample, ~0.5–1ug of DNA was sonicated to ~600 bp using an Episonic 1000E sonicator with 15‐s pulse intervals. Fragmentation was verified on a 1% agarose gel, and the process was repeated as necessary. Library preparation was conducted using a KAPA Hyper Library Prep Kit (KAPA Biosciences) using 10 bp TruSeq‐style oligonucleotide dual‐indexing barcodes (Faircloth & Glenn, 2012). Library preparation followed manufacturers protocols, with the exception that reaction sizes were scaled to 0.5×. Pre‐amplification and postlibrary amplification values were quantified before equimolar pooling of samples in batches of eight. A target capture approach was then used to amplify ultraconserved elements (UCEs; Faircloth et al., 2012). Pooled libraries were enriched for 1300 UCE loci using a custom probe set (Arbor Biosciences) originally designed by McGee et al. (2016), following manufacturers’ protocols. Pools were then amplified and cleaned using 16–18 PCR cycles following procedures outlined in Faircloth, Sorenson, Santini, and Alfaro (2013). These pools were then combined in equimolar ratios, and paired‐end fragments of 150 bp were sequenced on a single lane of an Illumina HiSeq Sequencer at the University of Oklahoma Medical Research Institute.

The sequenced libraries were demultiplexed, and barcodes, low‐quality base calls, and reads shorter than 40 bp were removed using Trimmomatic (Bolger, Lohse, & Usadel, 2014) as part of the program Illumiprocessor (Faircloth, 2013). Sequences were then assembled into de novo contigs using Trinity 2.0.6 with default parameters (Grabherr et al., 2011), and these were mapped to UCE probes using the Phyluce 1.5 pipeline (Faircloth, 2015). Sequence data were then processed in two ways optimized for phylogenomic or population genomic analyses.

For phylogenomic analyses, contigs were first aligned in the Phyluce pipeline using Mafft (Katoh & Standley, 2013) with the *no‐trim* option. Internal trimming using gblocks (Castresana, 2000) was then conducted on this alignment prior to outputting a final 70% complete data matrix. These alignments were then concatenated, and a maximum‐likelihood phylogenomic tree was then constructed using RAxML v8.1.24 (Stamatakis, 2014) on the CIPRES scientific gateway portal (Miller, Pfeiffer, & Schwartz, 2010). Two samples of *P. biafraensis *were included as outgroups for rooting the tree, as a previous study indicates this is the sister clade to the TEP species (Ludt et al., 2015). All analyses were completed using the GTRGAMMA model for bootstrapping, with 1,000 bootstrap iterations, and the rapid bootstrapping option (−*x*) selected. All nodes with a bootstrap value <50 were then collapsed.

Meanwhile, for the population genomic analyses, a reference dictionary was created to assist in SNP alignment using Picard (http://broadinstitute.github.io/picard/). This dictionary was created using the sample that recovered the most UCE loci. The reference was then indexed using SAMtools (Li et al., 2009). All samples were then aligned to this reference using BWA (Li & Durbin, 2009), using the maximal exact matches (MEM) command, with two threads, and the M option for downstream Piccard compatibility. Outputs were converted to BAM formats using SAMtools. The software Piccard was used for trimming, adding reading groups, and removing duplicated reads. All alignments were then merged, and sequences were re‐aligned around indels using the indel realigner function of the genome analysis toolkit (GATK; McKenna et al., 2010). Indels were then called and masked, and SNPs with a quality score above Q30 were kept and outputted to a VCF file using the variant filtration function in GATK. Low‐frequency alleles were removed from the dataset with a minor allele frequency value of 0.02. In order to minimize the influence of linkage disequilibrium in our statistical estimates, only one randomly chosen SNP per UCE locus was kept for all subsequent analyses. The resulting file was then converted to various formats for downstream analyses using the scripts of the seqcap_pop pipeline (https://github.com/mgharvey/seqcap_pop/).

A discriminant analysis of principal components (DAPC) was conducted to identify clusters in the SNP data with the package *adegenet* in R (Jombart, Devillard, & Balloux, 2010). This was conducted both with, and without outgroup samples of *P. biafraensis*. Since a DAPC that supports a single group cannot be graphed, the UCE SNP data was also examined with a principal component analysis (PCA) using the dudi.PCA command in the R package ade4 (Dray & Dufour, 2007). The program STRUCTURE v2.3.4 (Pritchard, Stephens, & Donnelly, 2000) was used to assign, and assess the fit of individuals to predetermined numbers of populations (*K*). An admixture model was used with correlated allele frequencies and no a priori populations information was given. Populations ranging between one and five (*K* = 1–5) were tested using 500,000 MCMC iterations after a burn‐in of 25,000. Five replicates were performed for each *K* to ensure convergence. Results were summarized with Structure Harvester (Earl, 2012) using the Evanno method (Evanno, Regnaut, & Goudet, 2005). Summary statistics of population genomic parameters (*F*_ST_, observed and expected heterozygosity, effective number of alleles, and Hardy–Weinberg equilibrium) were calculated using GenoDive v2 (Meirmans & Van Tienderen, 2004). An AMOVA was performed with 1,000 permutations to test for genetic structure between the two species, as well as between all sampling locations in GenoDive. The package PEGAS (Paradis, 2010) was used to examine the distribution of *F*_ST_ values across all loci in the dataset containing a single SNP per UCE locus, as well as across all SNPs.

### 2.5 Ecological comparisons

Considering the broad geographic range occupied by these sister species, it is quite possible that they are occupying ecologically distinct habitats, which could promote divergence even in the presence of gene flow (Bernardi, 2013; Rocha & Bowen, 2008; Rocha, Robertson, Roman, & Bowen, 2005). To test this, niche equivalency and similarity tests were conducted to determine if these two species are occupying similar habitats in the TEP (Broennimann et al., 2012). This approach uses kernel density smoothing to compare the density of species occurrence in environmental space using occurrence and environmental data. Occurrence data for both species was acquired from the Global Biodiversity Information Facility (GBIF) using the R package RGBIF (Chamberlain et al., 2017). Locality information was checked manually for errors, verifying species assignments with vouchered museum specimens or photographs. Eleven environmental layers that summarize bathymetry and annual properties of sea surface salinity (SSS) and sea surface temperature (SST) for the TEP were downloaded from the MARSPEC database (Sbrocco & Barber, 2013; http://www.marspec.org). These included: distance to shore, depth, mean annual range, and annual variance of SSS and SST, as well as the SSS of the wettest and driest months, and SST of the coldest and warmest month of the year. The comparison tests used here are bivariate, thus a principal components analysis was conducted using all 11 environmental layers, and the top two axes were kept for subsequent analyses. Niche equivalency and similarity tests were conducted in the R package ENMTools (Warren, Glor, & Turelli, 2010).

## 3 RESULTS

### 3.1 Phenotypic and morphological data

In total 169 vouchered museum specimens (103 *P. punctatus* specimens, 66 *P. laticlavius *specimens) were examined from the Scripps Institute of Oceanography, Natural History Museum of Los Angeles County, California Academy of Sciences, and Louisiana State University Museum of Natural Sciences. This included specimens distributed from across the entire TEP, including offshore islands (Supporting information Appendix S1: Table S1).

Overall, type specimens exhibited spotting patterns that were in agreement with the literature records of “pure” individuals (i.e., those without intermediate phenotypic traits). However, eight of the measured specimens had an intermediate phenotype of faint dark spots, suggesting a possible lack of reproductive isolation between the two groups or variation in squamation patterns (Supporting information Appendix S1: Figures S1, S2). These specimens mainly came from Costa Rica where the two species overlap. However, intermediate phenotypes were also found in Panama. Further, our morphological observations suggest all meristic counts and measurements overlapped for the two species. Dorsal‐fin rays were VII–VIII, 24–28, anal‐fin rays II–III, 22–24, and pectoral‐fin rays were 15–17 for both species. Body depth ranged from 1.6–2.1, pre‐dorsal‐fin length was 2.4–4.3, pre‐pelvic‐fin length was 2.2–3.6, and pre‐anal‐fin length was 1.3–3 in standard length for both species. The only perceivable difference was the modal number of pectoral‐fin (16 in *P. punctatus *and 17 in *P. laticlavius*) and dorsal‐fin rays (27 in *P. punctatus*, and 26 in *P. laticlavius*), but the ranges of these counts overlapped between the two species (Table 1).

**Table 1:** Averages and ranges of meristic and morphological measurements of the two species

	*P. punctatus*	*P. laticlavius*
Dorsal‐fin spines	VIII (VII–VIII)	VIII (VII–VIII)
Dorsal‐fin rays	27 (25–28)	26 (24–28)
Pectoral‐fin rays	16 (15–17)	17 (15–17)
Anal‐fin spines	III (II–III)	III (II–III)
Anal‐fin rays	23 (22–24)	23 (22–24)
Predorsal length	3.2 (2.4–4.3)	3.3 (2.5–4.2)
Prepelvic length	2.9 (2.4–3.6)	3 (2.2–3.7)
Pre‐anal length	2 (1.8–2.7)	2 (1.3–3)
Body depth	1.8 (1.6–2.1)	1.9 (1.6–2.1)

Notes

All morphological measurements are in comparison with standard length. Modes are reported for meristic counts, and means are reported for measurement comparisons.

### 3.2 Mitochondrial COI and sampling

In total, 53 individuals were collected, including 25 *P. punctatus*, 23 *P. laticlavius*, and 5 individuals with intermediate phenotypes that had faint spots restricted to certain regions of their bodies. The analyses reported here used all collected individuals, including fishes with intermediate phenotypes (the presence or absence of intermediates did not change the observed results).

**Figure 2:**
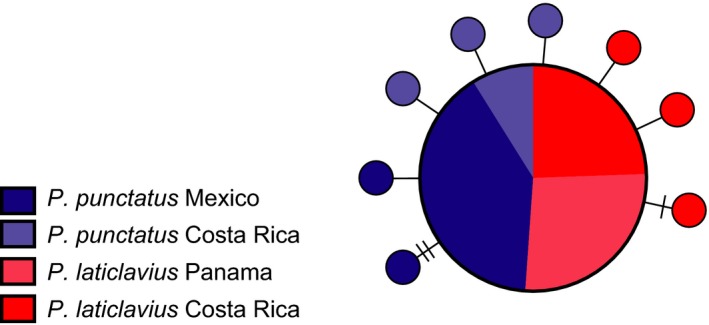
Mitochondrial COI haplotype network for both species of *Prionurus* across all sampling sites. Each circle represents a unique haplotype, and the size of the circle corresponds to the number of individuals that have that haplotype. Perpendicular dashes on connecting lines represent missing haplotypes

A portion of the mitochondrial COI gene (546 bp) was successfully amplified for all individuals. Regardless of how the data were analyzed, all results revealed low haplotype and nucleotide diversity. In total, nine haplotypes were recovered: one main haplotype shared between 45 individuals and eight singleton haplotypes (Figure 2). There was no genetic structure between either species or between any of the localities (Φ_ST_ = 0, for all comparisons). Furthermore, Fu's *F* statistic was negative in all comparisons (*F* = −9.1, *p < *0.001 for all samples; *F* = −4, *p* = 0.001 for *P. punctatus*; *F* = −2.5, *p = *0.006 for *P. laticlavius*). Overall, haplotype diversity was 0.282 for all samples and was 0.342 for *P. punctatus* and 0.222 for *P. laticlavius*, while nucleotide diversity was 0.001 for all comparisons. All COI summary statistics can be found in Table 2, and all sequences have been uploaded to GenBank under the accession numbers MK512611–MK512663.

**Table 2:** Mitochondrial DNA (COI) summary statistics for phenotypic groups and collection sites

Grouping	*N*	*N_h_*	*h*	π	Fu's *F*
By species					
*P. punctatus*	27	6	0.342 ± 0.117	0.001 ± 0.001	−3.965^a^
*P. laticlavius*	26	4	0.222 ± 0.106	0.001 ± 0.001	−2.451^a^
By locality					
Mexico	20	3	0.195 ± 0.15	0.001 ± 0.001	−0.626
Costa Rica	21	7	0.5 ± 0.133	0.001 ± 0.001	−5.074^a^
Panama	12	1	0	0	NA
Total	53	9	0.282 ± 0.082	0.001 ± 0.001	−9.099^a^

Note

Number of individuals (*N*), number of haplotypes (*N_h_*), haplotype diversity (*h*), nucleotide diversity (π), and Fu's F are given for each type of group.

a Significant *p‐*values (*p < *0.02; Fu, 1997).

### 3.3 UCE phylogenomics and population genomics

**Figure 3:**
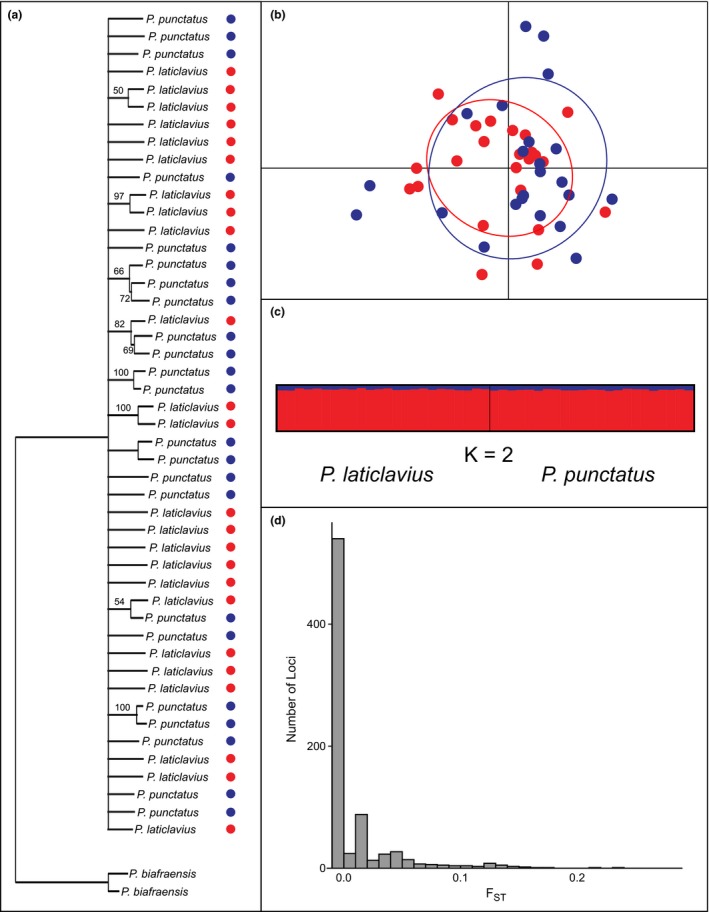
Summary of nuclear UCE results. Maximum‐likelihood phylogeny inferred from 866 concatenated loci, with nodes collapsed that have a bootstrap support <50 (a). Principal components plot with ellipses representing 95% confidence intervals (b). The most likely STRUCTURE clustering result (c). Distribution of locus‐by‐locus *F*_ST_ analyses (d). For A and B, blue represents *P. punctatus*, and red represents *P. laticlavius*

UCEs were successfully sequenced for 49 individuals: 23 *P. punctatus*, 24 *P. laticlavius*, as well as two individuals of *P. biafraensis *used as outgroups. The average number of sequencing reads per individual was 2.8 million and ranged from ~941,000–4.7 million. A data matrix with a completeness of 70% was assembled for phylogenomic analyses, which contained 866 UCE loci, with an average UCE locus length of 963 bp. The resulting phylogenomic hypothesis failed to recover the two species as reciprocally monophyletic, with overall low support throughout the tree (Figure 3a).

Meanwhile, after filtering, the population genomic approach identified a total of 8,757 SNPs in the data, which was reduced to 864 SNP‐loci after randomly selecting a single SNP per UCE locus. These SNPs had an average sequence depth of 30x coverage. The AMOVA found significant, albeit low, structuring between the two species (*F*_ST_ = 0.013, *p < *0.001). If genetic variation is examined by sampling location, significant structuring is found between Mexico and all other locations (*F*_ST_ = 0.014, *p < *0.001 for Costa Rica comparison, and *F*_ST_ = 0.018, *p < *0.001 for Panama comparison). However, no significant structure was found between Costa Rica and Panama (*F*_ST_ = 0.003, *p = *0.198). All pairwise comparisons can be found in Table 3.

**Table 3:** Pairwise comparisons between species and locations for mtDNA COI (Φ_ST_ values reported below diagonal) and UCE SNPs (*F*_ST_ values reported above diagonal)

	By species			By locality		
	*P. punctatus*	*P. laticlavius*		Mexico	Costa Rica	Panama
*P. punctatus*	–	0.013^a^	Mexico	–	0.014^a^	0.018^a^
*P. laticlavius*	0	–	Costa Rica	0	–	0.003
			Panama	0	0	–

a Significant AMOVA *p*‐values (*p* ≤ 0.05).

DAPC analyses that included *P. biafraensis* suggested the most likely number of clusters to be two, with the sister‐species pair *P. punctatus* and *P. laticlavius* together in a single group. This pattern could be driven by large genetic divergence between *P. biafraensis* and both TEP species, which could mask any subtle differences between the two TEP species. However, when the outgroup *P. biafraensis* is removed, the most likely number of clusters recovered is one, with both TEP species clustering together. This result can also be seen in a PCA of the SNP dataset, which reveals both species completely overlapping in 95% confidence intervals (Figure 3b). These results are mirrored by our STRUCTURE analyses. When testing between *K = *1–5, a comparison of model outputs with the Evanno method recovered *K = *2 as the most likely result, with *K = *1 the second most likely number of clusters (Supporting information Appendix S1: Table S2). However, the two clusters recovered do not correspond to the two TEP species, but rather differences in allele frequencies for particular sets of loci (Figure 3c). Examining the distribution of individual locus *F*_ST_ values further reveals little to no divergence between the species. Most comparisons resulted in *F*_ST_ = 0, with the highest divergence for a locus being *F*_ST_ = 0.24 (Figure 3d). Even when the analyses were expanded to include all 8,757 SNPs, no single locus was found to be alternatively fixed between the two species. Furthermore, while results slightly vary when repeating all analyses with different sets of randomly selected SNPs for each UCE locus, the overall conclusions remain consistent. Raw reads and assembled UCE loci for all individuals are deposited on GenBank under the project number PRJNA516931.

### 3.4 Ecological Niche models

After accounting for duplicates and filtering questionable locality points, we recovered 86 occurrence points for *P. punctatus* and 50 occurrence points for *P. laticlavius*. The PCA of the 11 environmental layers found that PC1 encompasses 48% of the environmental variation in these layers, and that PC2 encompasses 23% of remaining variation, together totaling ~71% of all variation in the environmental layers. Comparisons of niche equivalency and similarity both are concurrent with the null hypothesis that these species are occupying equivalent habitats (all *p *values >0.05; Supporting information Appendix S1: Figure S3)

### 3.5 Systematic status of *Prionurus punctatus* Gill 1862

Morphological features distinguishing *Prionurus punctatus *from *P. laticlavius *are inconsistent and not related to any genetic relationships of distinct populations. Without any basis for recognizing these taxa as distinct, we formally recognize *Prionurus punctatus* Gill, 1862 as a junior subjective synonym of *P. laticlavius *(Valenciennes 1846).

## 4 DISCUSSION

Slight differences in color patterns between populations can suggest that such groups are following distinct evolutionary trajectories. However, even consistent differences in color patterns can sometimes be misleading, as contrasting phenotypes do not always correspond to distinct genetic clusters. The results from our study suggest that highly vagile *Prionurus* surgeonfishes in the TEP are a clear example of this paradox: two taxa that have been recognized as distinct species for over 150 years by the presence or absence of dark spots show no consistent morphological or genomic divergence.

This study represents the most comprehensive morphological analysis for *P. laticlavius *(including the former *P. punctatus*), as it includes historical specimens of both phenotypes, as well as individuals collected from offshore islands (Galapagos and the Revillagigedos). Overall, our results are very clear in showing complete overlap of all meristic counts and measurements between the two phenotypes. Perhaps the most unique observation is that the spotting pattern is not discrete, as suggested by the type specimens of these species. Several individuals display faint spots on parts of their bodies (Supporting information Appendix S1: Figure S1), and while these phenotypic traits could be interpreted as evidence of hybridization without any other information, the lack of any genetic structuring between the species suggests that this is merely an intermediate phenotype between two populations.

Mitochondrial analyses revealed a single main haplotype distributed across the entire coastline of the TEP, resulting in low haplotype and nucleotide diversities. This genetic signature is typically observed in groups that have recently experienced a population bottleneck, or recent founder events (Grant & Bowen, 1998). A founder event seems unlikely given that the TEP *Prionurus* are the sister group to *P. biafraensis *from the eastern Atlantic and must have had a common ancestor in the Central American Seaway prior to the closure of the Isthmus of Panama (Ludt et al., 2015). However, it is reasonable to expect that this group recently underwent a population bottleneck. Using fossil calibrations, Ludt et al. (2015) estimated a crown age for the TEP *Prionurus* in the late Pleistocene, ~490,000 years before present (95% HPD intervals ranging from 70,000 years ago–1.2 million years ago). This divergence estimate is contemporary with the climatic shifts promoted by the Pleistocene glaciations, which impacted many other marine organisms in a similar way (Ludt & Rocha, 2015). These climatic shifts also correspond with the appearance of upwelling areas and ENSO oscillations in the TEP (Cortes, 1997; Cortés, 2003). All of these changes contributed to a period of rapid community turnover in the reef structure of the TEP from a community composed of Atlantic‐related corals, to a community of sparsely distributed Pacific corals (Leigh, O'Dea, & Vermeij, 2014; López‐Pérez, 2017). This turnover could easily result in population fluctuations and potential population bottlenecks.

In our comparison of nuclear loci, which are gathered from SNPs distributed throughout the entire genome, a similar pattern of little to no differentiation between the phenotypes was recovered. This dataset failed to reveal any alternatively fixed alleles between the two phenotypes. However, a low, but significant, *F*_ST_ was found between spotted and nonspotted individuals. This value was comparable to *F*_ST_ estimates of different collection sites (e.g., Mexico vs. Central American localities), and these results suggest a possible signature of isolation by distance, which has been previously reported for other fishes of the TEP (Bernal, Gaither, Simison, & Rocha, 2017; Lessios & Baums, 2017). It would be tempting to suggest that SNPs gathered from UCEs lack sufficient signal to detect differentiation at this time scale given the conserved nature of these genomic regions. However, only the cores of these loci are conserved, and variation increases in the regions flanking this core (Faircloth et al., 2012; Gilbert et al., 2015). In fact, SNPs gathered from UCEs have been proven informative in detecting population structure at shallow timescales for various taxa (e.g., in birds: Harvey, Aleixo, Ribas, & Brumfield, 2017; Oswald et al., 2016; Smith, Harvey, Faircloth, Glenn, & Brumfield, 2013, and fishes: Burress et al., 2018). Thus, it is likely that the similarities between the mitochondrial and UCE loci reflect an actual shared history, and that this situation echoes one in which a single species displays color variation across its range.

We compared the abiotic habitats that these phenotypic variants occupy to test whether ecology could be a driving factor in the divergence of these two groups. Using locality data from across the entire range of this species, we failed to detect any significant differences in the abiotic habitats that the two phenotypes occupy. However, these data are all associated with the abiotic habitat of the region (e.g., temperature, salinity), and they do not take into account any biotic factors (e.g., coral cover, species interactions, productivity), which could differ throughout the range of the focal species. Despite this limitation, *Prionurus* appears to traverse multiple ecotypes of the region. This is well illustrated by the Central American faunal gap, an ~1000km stretch of coastal habitat lacking coral reef ecosystems that has been suggested as a barrier between the Mexican and Panamic provinces of the TEP, influencing the connectivity and distribution of multiple species (Briggs, 1974; Hastings, 2000; Robertson & Cramer, 2009; Springer, 1959). The two phenotypes of *P. laticlavius* are roughly separated by this gap, however, the spotted phenotype does occur further south, suggesting this habitat discontinuity is not sufficient for restricting gene flow along the continental coast.

This study adds to the growing list of examples where differences in phenotype are not accompanied by genetic structure. Examples of this in reef fishes can be found in angelfishes (DiBattista et al., 2012; Schultz, Pyle, DeMartini, & Bowen, 2007), butterflyfishes (DiBattista et al., 2015), damselfishes (Leray et al. 2010), groupers (Craig et al., 2006), and Caribbean hamlets (McCartney et al. 2003; Ramon et al., 2003; Garcia‐Machado, Monteagudo, & Solignac, 2004) among others. The latter is perhaps the most well‐studied example for reef fishes, where 11 distinct color phenotypes exist in a genetically homogeneous species complex (Puebla, Bermingham, & Guichard, 2008). Genome scans have thus far only detected a single outlier locus, which corresponds to a *Hox *gene that could be associated with differences in coloration (Puebla, Bermingham, & McMillan, 2014). Something similar could be taking place in *Prionurus*, where slight differences in squamation patterns could be controlled by a small number of alternatively fixed loci. However, since these genomic regions were not detected with our targeted capture approach, this hypothesis remains elusive.

While this study found a lack of divergence among two TEP surgeonfishes, it does give insight into the evolutionary processes that can take place in the region. Pleistocene glaciations resulted in the whole‐scale community turnover of corals in the TEP, which may have adversely impacted all reef‐dwelling species (López‐Pérez, 2017). This study shows that a prominent, large‐bodied, schooling herbivore underwent a dramatic population bottleneck recently, possibly as a result of TEP environmental fluctuations during and after the closure of the Isthmus of Panama. A scenario where a severe population bottleneck results in several distant, small populations could lead to fixing of alternative spotting patterns in this surgeonfish, which can be rapidly fixed through genetic drift. In this case, incomplete dominance at a single locus could explain the prevalence of intermediate phenotypes, and this scenario could also explain the modal differences observed in the pectoral‐fin and dorsal‐fin ray counts between the two phenotypes.

In species that are more dispersal limited, or that have more rapid turnover rates with shorter generation times, these environmental fluctuations and corresponding population bottlenecks could result in isolated populations that ultimately form new species, suggesting a mechanism in which TEP in situ speciation can occur in allopatry (Hastings, 2000). However, this study also highlights why in situ speciation along the TEP coastline may be uncommon in large‐bodied fishes, as these surgeonfishes are perhaps some of the best dispersers among reef fishes, and have long generations times (~45 years for other species of this genus; Choat & Axe, 1996) allowing populations to regain connectivity after population bottleneck events. Additionally, such severe population crashes could also easily result in high extinction rates, contributing to the reduced diversification rates previously observed for this region (Cowman & Bellwood 2013). Ultimately, an extended genomic approach that targets whole genomes, including samples from oceanic islands, could reveal the molecular underpinnings of the squamation patterns of *P. laticlavius*. Further studies including a diverse set of endemic taxa in the TEP are needed to shed light on how speciation occurs in one of the most distinctive tropical marine regions of the world.

## CONFLICT OF INTEREST

None declared.

## AUTHOR CONTRIBUTIONS

WBL and PC conceived of the study; WBL, MAB, and ES collected the samples; WBL and EK conducted all of the laboratory work; WBL, EK, and MAB analyzed the data; WBL wrote the manuscript; MAB, ES, and PC provided essential input into the manuscript; all authors approved of the final submission.

## Data Availability

COI sequences have been deposited on GenBank under the accession numbers MK512611–MK612663. Raw reads and assembled UCE loci have been deposited on GenBank under the project number PRJNA516931. Files containing niche model layers, locality data, and alignment files for COI and UCE data can be found at Dryad https://doi.org/10.5061/dryad.27js55v.

## References

[ece35029-bib-0001] Allen, G. R. , & Robertson, D. R. (1994). Fishes of the tropical eastern Pacific. Honolulu, HI: University of Hawaii Press.

[ece35029-bib-0002] Alva‐Campbell, Y. , Floeter, S. R. , Robertson, D. R. , Bellwood, D. R. , & Bernardi, G. (2010). Molecular phylogenetics and evolution of *Holacanthus* angelfishes (Pomacanthidae). Molecular Phylogenetics and Evolution, 56(1), 456–461. 10.1016/j.ympev.2010.02.014 20171293

[ece35029-bib-0003] Bernal, M. A. , Gaither, M. R. , Simison, W. B. , & Rocha, L. A. (2017). Introgression and selection shaped the evolutionary history of sympatric sister‐species of coral reef fishes (genus: *Haemulon*). Molecular Ecology, 26(2), 639–652. 27873385 10.1111/mec.13937

[ece35029-bib-0004] Bernardi, G. (2013). Speciation in fishes. Molecular Ecology, 22(22), 5487–5502. 10.1111/mec.12494 24118417

[ece35029-bib-0005] Bernardi, G. , Alva‐Campbell, Y. R. , Gasparini, J. L. , & Floeter, S. R. (2008). Molecular ecology, speciation, and evolution of the reef fish genus *Anisotremus* . Molecular Phylogenetics and Evolution, 48(3), 929–935. 10.1016/j.ympev.2008.05.011 18667336

[ece35029-bib-0006] Bolger, A. M. , Lohse, M. , & Usadel, B. (2014). Trimmomatic: A flexible trimmer for Illumina sequence data. Bioinformatics, 30(15), 2114–2120. 10.1093/bioinformatics/btu170 24695404 PMC4103590

[ece35029-bib-0007] Briggs, J. C. (1974). Marine zoogeography. New York, NY: McGraw Hill.

[ece35029-bib-0008] Briggs, J. C. , & Bowen, B. W. (2012). A realignment of marine biogeographic provinces with particular reference to fish distributions. Journal of Biogeography, 39(1), 12–30. 10.1111/j.1365-2699.2011.02613.x

[ece35029-bib-0009] Broennimann, O. , Fitzpatrick, M. C. , Pearman, P. B. , Petitpierre, B. , Pellissier, L. , Yoccoz, N. G. , … Guisan, A. (2012). Measuring ecological niche overlap from occurrence and spatial environmental data. Global Ecology and Biogeography, 21(4), 481–497. 10.1111/j.1466-8238.2011.00698.x

[ece35029-bib-0010] Burress, E. D. , Alda, F. , Duarte, A. , Loureiro, M. , Armbruster, J. W. , & Chakrabarty, P. (2018). Phylogenomics of pike cichlids (Cichlidae: *Crenicichla*): the rapid ecological speciation of an incipient species flock. Journal of evolutionary biology, 31(1), 14-30. 29044782 10.1111/jeb.13196

[ece35029-bib-0011] Castresana, J. (2000). Selection of conserved blocks from multiple alignments for their use in phylogenetic analysis. Molecular Biology and Evolution, 17(4), 540–552. 10.1093/oxfordjournals.molbev.a026334 10742046

[ece35029-bib-0012] Chamberlain, S. , Barve, V. , Mcglinn, D. , & Chamberlain, M. S. (2017). Package ‘rgbif’.

[ece35029-bib-0013] Choat, J. H. , & Axe, L. M. (1996). Growth and longevity in acanthurid fishes; an analysis of otolith increments. Marine Ecology Progress Series, 15–26. 10.3354/meps134015

[ece35029-bib-0014] Clement, M. , Posada, D. C. K. A. , & Crandall, K. A. (2000). TCS: A computer program to estimate gene genealogies. Molecular Ecology, 9(10), 1657–1659. 10.1046/j.1365-294x.2000.01020.x 11050560

[ece35029-bib-0015] Coleman, R. R. , Eble, J. A. , DiBattista, J. D. , Rocha, L. A. , Randall, J. E. , Berumen, M. L. , & Bowen, B. W. (2016). Regal phylogeography: Range-wide survey of the marine angelfish *Pygoplites diacanthus* reveals evolutionary partitions between the Red Sea, Indian Ocean, and Pacific Ocean. Molecular phylogenetics and evolution, 100, 243–253. 27068838 10.1016/j.ympev.2016.04.005

[ece35029-bib-0016] Cortes, J. (1997). Biology and geology of coral reefs of the eastern Pacific. Coral Reefs, 16(Suppl.), S39–S46.

[ece35029-bib-0017] Cortés, J. (2003). Coral reefs of the Americas: An introduction to Latin American Coral Reefs In Cortés J. (Ed.), Latin American Coral Reefs (pp. 361–385). Holland, The Netherlands: Elsevier Science.

[ece35029-bib-0018] Cortés, J. (2012). Marine biodiversity of an Eastern Tropical Pacific oceanic island, Isla del Coco, Costa Rica. Revista De Biología Tropical, 60, 131–185.

[ece35029-bib-0019] Cowman, P. F. , & Bellwood, D. R. (2013). The historical biogeography of coral reef fishes: Global patterns of origination and dispersal. Journal of Biogeography, 40(2), 209–224. 10.1111/jbi.12003

[ece35029-bib-0020] Craig, M. T. , Hastings, P. A. , Pondella, D. J. , Ross Robertson, D. , & Rosales‐Casián, J. A. (2006). Phylogeography of the flag cabrilla *Epinephelus labriformis* (Serranidae): Implications for the biogeography of the Tropical Eastern Pacific and the early stages of speciation in a marine shore fish. Journal of Biogeography, 33(6), 969–979. 10.1111/j.1365-2699.2006.01467.x

[ece35029-bib-0021] De Queiroz, K. (2005). Ernst Mayr and the modern concept of species. Proceedings of the National Academy of Sciences, 102, 6600–6607. 10.1073/pnas.0502030102 PMC1131873 15851674

[ece35029-bib-0022] DiBattista, J. D. , Berumen, M. L. , Gaither, M. R. , Rocha, L. A. , Eble, J. A. , Choat, J. H. , … Bowen, B. W. (2013). After continents divide: comparative phylogeography of reef fishes from the Red Sea and Indian Ocean. Journal of Biogeography, 40(6), 1170–1181.

[ece35029-bib-0023] DiBattista, J. D. , Waldrop, E. , Bowen, B. W. , Schultz, J. K. , Gaither, M. R. , Pyle, R. L. , & Rocha, L. A. (2012). Twisted sister species of pygmy angelfishes: Discordance between taxonomy, coloration, and phylogenetics. Coral Reefs, 31(3), 839–851. 10.1007/s00338-012-0907-y

[ece35029-bib-0024] DiBattista, J. D. , Waldrop, E. , Rocha, L. A. , Craig, M. T. , Berumen, M. L. , & Bowen, B. W. (2015). Blinded by the bright: A lack of congruence between colour morphs, phylogeography and taxonomy for a cosmopolitan Indo‐Pacific butterflyfish, *Chaetodon auriga* . Journal of Biogeography, 42(10), 1919–1929.

[ece35029-bib-0025] DiBattista, J. D. , Wilcox, C. , Craig, M. T. , Rocha, L. A. , & Bowen, B. W. (2011). Phylogeography of the Pacific Blueline Surgeonfish, *Acanthurus nigroris*, reveals high genetic connectivity and a cryptic endemic species in the Hawaiian Archipelago. Journal of Marine Biology, 2011, 4001–17.

[ece35029-bib-0026] Doherty, P. J. , Planes, S. , & Mather, P. (1995). Gene flow and larval duration in seven species of fish from the Great Barrier Reef. Ecology, 76(8), 2373–2391. 10.2307/2265814

[ece35029-bib-0027] Dray, S. , & Dufour, A. B. (2007). The ade4 package: Implementing the duality diagram for ecologists. Journal of Statistical Software, 22(4), 4001–20.

[ece35029-bib-0028] Earl, D. A. , & vonHoldt, B. M. (2012). STRUCTURE HARVESTER: A website and program for visualizing STRUCTURE output and implementing the Evanno method. Conservation Genetics Resources, 4(2), 359–361. 10.1007/s12686-011-9548-7

[ece35029-bib-0029] Eble, J. A. , Rocha, L. A. , Craig, M. T. , & Bowen, B. W. . (2011). Not all larvae stay close to home: insights into marine population connectivity with a focus on the brown surgeonfish (*Acanthurus nigrofuscus*). Journal of Marine Biology, 2011, 4001–4012. 10.1155/2011/518516 PMC4260469 25505914

[ece35029-bib-0030] Eble, J. A. , Toonen, R. J. , & Bowen, B. W. (2009). Endemism and dispersal: Comparative phylogeography of three surgeonfishes across the Hawaiian Archipelago. Marine Biology, 156(4), 689–698. 10.1007/s00227-008-1119-4

[ece35029-bib-0031] Evanno, G. , Regnaut, S. , & Goudet, J. (2005). Detecting the number of clusters of individuals using the software STRUCTURE: A simulation study. Molecular Ecology, 14(8), 2611–2620. 10.1111/j.1365-294X.2005.02553.x 15969739

[ece35029-bib-0032] Excoffier, L. , Laval, G. , & Schneider, S. (2005). Arlequin (version 3.0): An integrated software package for population genetics data analysis. Evolutionary Bioinformatics, 1, 117693430500100. PMC2658868 19325852

[ece35029-bib-0033] Eytan, R. I. , Hastings, P. A. , Holland, B. R. , & Hellberg, M. E. (2012). Reconciling molecules and morphology: Molecular systematics and biogeography of Neotropical blennies (*Acanthemblemaria*). Molecular Phylogenetics and Evolution, 62(1), 159–173. 10.1016/j.ympev.2011.09.028 22040767

[ece35029-bib-0034] Faircloth, B. C. (2013). Illumiprocessor: A Trimmomatic wrapper for parallel adapter and quality trimming. 10.6079/J9ILL

[ece35029-bib-0035] Faircloth, B. C. (2015). PHYLUCE is a software package for the analysis of conserved genomic loci. Bioinformatics, 32(5), 786–788. 10.1093/bioinformatics/btv646 26530724

[ece35029-bib-0036] Faircloth, B. C. , & Glenn, T. C. (2012). Not all sequence tags are created equal: Designing and validating sequence identification tags robust to indels. PLoS ONE, 7(8), e42543 10.1371/journal.pone.0042543 22900027 PMC3416851

[ece35029-bib-0037] Faircloth, B. C. , McCormack, J. E. , Crawford, N. G. , Harvey, M. G. , Brumfield, R. T. , & Glenn, T. C. (2012). Ultraconserved elements anchor thousands of genetic markers spanning multiple evolutionary timescales. Systematic Biology, 61(5), 717–726. 10.1093/sysbio/sys004 22232343

[ece35029-bib-0038] Faircloth, B. C. , Sorenson, L. , Santini, F. , & Alfaro, M. E. (2013). A phylogenomic perspective on the radiation of ray‐finned fishes based upon targeted sequencing of ultraconserved elements (UCEs). PLoS ONE, 8(6), e65923 10.1371/journal.pone.0065923 23824177 PMC3688804

[ece35029-bib-0039] Fu, Y. X. (1997). Statistical tests of neutrality of mutations against population growth, hitchhiking and background selection. Genetics, 147(2), 915–925. 9335623 10.1093/genetics/147.2.915 PMC1208208

[ece35029-bib-0040] Gaither, M. R. , Bernal, M. A. , Coleman, R. R. , Bowen, B. W. , Jones, S. A. , Simison, W. B. , & Rocha, L. A. (2015). Genomic signatures of geographic isolation and natural selection in coral reef fishes. Molecular Ecology, 24(7), 1543–1557. 25753379 10.1111/mec.13129

[ece35029-bib-0041] Gaither, M. R. , Schultz, J. K. , Bellwood, D. R. , Pyle, R. L. , DiBattista, J. D. , Rocha, L. A. , & Bowen, B. W. (2014). Evolution of pygmy angelfishes: Recent divergences, introgression, and the usefulness of color in taxonomy. Molecular Phylogenetics and Evolution, 74, 38–47. 10.1016/j.ympev.2014.01.017 24500654

[ece35029-bib-0042] Garcia‐Machado, E. , Monteagudo, P. C. , & Solignac, M. (2004). Lack of mtDNA differentiation among hamlets (*Hypoplectrus*, Serranidae). Marine Biology, 144(1), 147–152. 10.1007/s00227-003-1174-9

[ece35029-bib-0043] Gilbert, P. S. , Chang, J. , Pan, C. , Sobel, E. M. , Sinsheimer, J. S. , Faircloth, B. C. , & Alfaro, M. E. (2015). Genome‐wide ultraconserved elements exhibit higher phylogenetic informativeness than traditional gene markers in percomorph fishes. Molecular Phylogenetics and Evolution, 92, 140–146. 10.1016/j.ympev.2015.05.027 26079130 PMC4583375

[ece35029-bib-0044] Gill, T. N. (1862). Catalogue of the fishes of Lower California, in the Smithsonian Institution, collected by Mr. J. Xantus. Part II. Proceedings of the Academy of Natural Sciences of Philadelphia, 14, 242–246.

[ece35029-bib-0045] Grabherr, M. G. , Haas, B. J. , Yassour, M. , Levin, J. Z. , Thompson, D. A. , Amit, I. , … Chen, Z. (2011). Trinity: Reconstructing a full‐length transcriptome without a genome from RNA‐Seq data. NatureBiotechnology, 29(7), 644. 10.1038/nbt.1883 PMC3571712 21572440

[ece35029-bib-0046] Grant, W. A. S. , & Bowen, B. W. (1998). Shallow population histories in deep evolutionary lineages of marine fishes: Insights from sardines and anchovies and lessons for conservation. Journal of Heredity, 89(5), 415–426. 10.1093/jhered/89.5.415

[ece35029-bib-0047] Harvey, M. G. , Aleixo, A. , Ribas, C. C. , & Brumfield, R. T. (2017). Habitat association predicts genetic diversity and population divergence in Amazonian Birds. The American Naturalist, 190(5), 631–648. 10.1086/693856 29053360

[ece35029-bib-0049] Hastings, P. A. (2000). Biogeography of the tropical eastern Pacific: Distribution and phylogeny of chaenopsid fishes. Zoological Journal of the Linnean Society, 128(3), 319–335. 10.1111/j.1096-3642.2000.tb00166.x

[ece35029-bib-0051] Jombart, T. , Devillard, S. , & Balloux, F. (2010). Discriminant analysis of principal components: A new method for the analysis of genetically structured populations. BMC Genetics, 11(1), 94 10.1186/1471-2156-11-94 20950446 PMC2973851

[ece35029-bib-0052] Katoh, K. , & Standley, D. M. (2013). MAFFT multiple sequence alignment software version 7: Improvements in performance and usability. Molecular Biology and Evolution, 30(4), 772–780. 10.1093/molbev/mst010 23329690 PMC3603318

[ece35029-bib-0053] Leigh, E. G. , O'Dea, A. , & Vermeij, G. J. (2014). Historical biogeography of the Isthmus of Panama. Biological Reviews, 89, 148–172. 23869709 10.1111/brv.12048

[ece35029-bib-0054] Leray, M. , Beldade, R. , Holbrook, S. J. , Schmitt, R. J. , Planes, S. , & Bernardi, G. (2010). Allopatric divergence and speciation in coral reef fish: The three‐spot *Dascyllus, Dascyllus trimaculatus*, species complex. Evolution, 64(5), 1218–1230. 20002167 10.1111/j.1558-5646.2009.00917.x

[ece35029-bib-0055] Lessios, H. A. , & Baums, I. B. (2017). Gene flow in coral reef organisms of the tropical eastern Pacific In Coral reefs of the eastern Tropical Pacific (pp. 477–499). Dordrecht, The Netherlands: Springer.

[ece35029-bib-0056] Li, H. , & Durbin, R. (2009). Fast and accurate short read alignment with Burrows‐Wheeler transform. Bioinformatics, 25(14), 1754–1760. 10.1093/bioinformatics/btp324 19451168 PMC2705234

[ece35029-bib-0057] Li, H. , Handsaker, B. , Wysoker, A. , Fennell, T. , Ruan, J. , Homer, N. , … Durbin, R. (2009). The sequence alignment/map format and SAMtools. Bioinformatics, 25(16), 2078–2079. 10.1093/bioinformatics/btp352 19505943 PMC2723002

[ece35029-bib-0058] Lin, H. C. , Sanchez-Ortiz, C. , & Hastings, P. A. (2009). Colour variation is incongruent with mitochondrial lineages: cryptic speciation and subsequent diversification in a Gulf of California reef fish (Teleostei: Blennioidei). Molecular Ecology, 18(11), 2476–2488. 19389167 10.1111/j.1365-294X.2009.04188.x

[ece35029-bib-0059] Lin, H. C. , & Hastings, P. A. (2011). Evolution of a Neotropical marine fish lineage (Subfamily Chaenopsinae, Suborder Blennioidei) based on phylogenetic analysis of combined molecular and morphological data. Molecular Phylogenetics and Evolution, 60(2), 236–248. 10.1016/j.ympev.2011.04.018 21550409

[ece35029-bib-0060] López‐Pérez, A. (2017). Revisiting the Cenozoic History and the Origin of the Eastern Pacific Coral Fauna In Coral Reefs of the Eastern Tropical Pacific (pp. 39–57). Dordrecht, The Netherlands: Springer Netherlands.

[ece35029-bib-0061] Ludt, W. B. , & Rocha, L. A. (2015). Shifting seas: The impacts of Pleistocene sea‐level fluctuations on the evolution of tropical marine taxa. Journal of Biogeography, 42(1), 25–38. 10.1111/jbi.12416

[ece35029-bib-0062] Ludt, W. B. , Rocha, L. A. , Erdmann, M. V. , & Chakrabarty, P. (2015). Skipping across the tropics: The evolutionary history of sawtail surgeonfishes (Acanthuridae: *Prionurus*). Molecular Phylogenetics and Evolution, 84, 166–172. 10.1016/j.ympev.2014.12.017 25596541

[ece35029-bib-0063] McCartney, M. A. , Acevedo, J. , Heredia, C. , Rico, C. , Quenoville, B. , Bermingham, E. , & McMILLAN, W. O. (2003). Genetic mosaic in a marine species flock. Molecular Ecology, 12(11), 2963–2973. 14629377 10.1046/j.1365-294x.2003.01946.x

[ece35029-bib-0064] McGee, M. D. , Faircloth, B. C. , Borstein, S. R. , Zheng, J. , Hulsey, C. D. , Wainwright, P. C. , & Alfaro, M. E. (2016). Replicated divergence in cichlid radiations mirrors a major vertebrate innovation. Proceedings of the Royal Society of London B, 283(1822), 20151413 10.1098/rspb.2015.1413 PMC4721080 26763694

[ece35029-bib-0065] McKenna, A. , Hanna, M. , Banks, E. , Sivachenko, A. , Cibulskis, K. , Kernytsky, A. , … DePristo, M. a. (2010). The Genome Analysis Toolkit: A MapReduce framework for analyzing next‐generation DNA sequencing data. GenomeResearch, 20(9), 1297–1303. 10.1101/gr.107524.110 PMC2928508 20644199

[ece35029-bib-0067] Meirmans, P. G. , & Van Tienderen, P. H. (2004). GENOTYPE and GENODIVE: Two programs for the analysis of genetic diversity of asexual organisms. Molecular Ecology Notes, 4(4), 792–794. 10.1111/j.1471-8286.2004.00770.x

[ece35029-bib-0068] Miller, E. C. , Lin, H. C. , & Hastings, P. A. (2016). Improved resolution and a novel phylogeny for the Neotropical triplefin blennies (Teleostei: Tripterygiidae). Molecular Phylogenetics and Evolution, 96, 70–78. 10.1016/j.ympev.2015.12.003 26718057

[ece35029-bib-0069] Miller, M. A. , Pfeiffer, W. , & Schwartz, T. (2010). Creating the CIPRES Science Gateway for inference of large phylogenetic trees In Gateway Computing Environments Workshop (GCE), 2010 (pp. 4001–8). IEEE.

[ece35029-bib-0070] Oswald, J. A. , Harvey, M. G. , Remsen, R. C. , Foxworth, D. U. , Cardiff, S. W. , Dittmann, D. L. , … Brumfield, R. T. (2016). Willet be one species or two? A genomic view of the evolutionary history of *Tringa semipalmata* . The Auk, 133(4), 593–614.

[ece35029-bib-0071] Paradis, E. (2010). pegas: An R package for population genetics with an integrated–modular approach. Bioinformatics, 26(3), 419–420. 10.1093/bioinformatics/btp696 20080509

[ece35029-bib-0074] Pritchard, J. K. , Stephens, M. , & Donnelly, P. (2000). Inference of population structure using multilocus genotype data. Genetics, 155(2), 945–959. 10835412 10.1093/genetics/155.2.945 PMC1461096

[ece35029-bib-0075] Puebla, O. , Bermingham, E. , & Guichard, F. (2008). Population genetic analyses of *Hypoplectrus* coral reef fishes provide evidence that local processes are operating during the early stages of marine adaptive radiations. Molecular Ecology, 17(6), 1405–1415. 10.1111/j.1365-294X.2007.03654.x 18321253

[ece35029-bib-0076] Puebla, O. , Bermingham, E. , & McMillan, W. O. (2014). Genomic atolls of differentiation in coral reef fishes (*Hypoplectrus spp*., Serranidae). Molecular Ecology, 23(21), 5291–5303. 25231270 10.1111/mec.12926

[ece35029-bib-0077] Ramon, M. L. , Lobel, P. S. , & Sorenson, M. D. (2003). Lack of mitochondrial genetic structure in hamlets (*Hypoplectrus spp*.): Recent speciation or ongoing hybridization? Molecular Ecology, 12(11), 2975–2980. 14629378 10.1046/j.1365-294x.2003.01966.x

[ece35029-bib-0078] Randall, J. E. (2001). Surgeonfishes of Hawai'i and the world. Honolulu, HI: Bishop Museum Press.

[ece35029-bib-0079] Randall, J. E. , & Rocha, L. A. (2009). *Halichoeres claudia* sp. nov., a new Indo‐Pacific wrasse (Perciformes: Labridae), the fourth species of the *H. ornatissimus* complex. Zoological Studies, 48, 709–718.

[ece35029-bib-0080] Riginos, C. (2005). Cryptic vicariance in Gulf of California fishes parallels vicariant patterns found in Baja California mammals and reptiles. Evolution, 59(12), 2678–2690. 10.1111/j.0014-3820.2005.tb00979.x 16526514

[ece35029-bib-0081] Robertson, D. R. , & Allen, G. R. (2015). Shorefishes of the Tropical Eastern Pacific: Online information system. Version 2.0 Smithsonian Tropical. Balboa, Panamá: Research Institute.

[ece35029-bib-0082] Robertson, D. R. , & Cramer, K. L. (2009). Shore fishes and biogeographic subdivisions of the Tropical Eastern Pacific. Marine Ecology Progress Series, 380, 4001–17. 10.3354/meps07925

[ece35029-bib-0083] Rocha, L. A. (2004). Mitochondrial DNA and color pattern variation in three western Atlantic *Halichoeres* (Labridae), with the revalidation of two species. Copeia, 2004(4), 770–782. 10.1643/CG-04-106

[ece35029-bib-0084] Rocha, L. A. , & Bowen, B. W. (2008). Speciation in coral‐reef fishes. Journal of Fish Biology, 72(5), 1101–1121. 10.1111/j.1095-8649.2007.01770.x

[ece35029-bib-0085] Rocha, L. A. , Lindeman, K. C. , Rocha, C. R. , & Lessios, H. A. (2008). Historical biogeography and speciation in the reef fish genus *Haemulon *(Teleostei: Haemulidae). Molecular Phylogenetics and Evolution, 48(3), 918–928. 10.1016/j.ympev.2008.05.024 18599320

[ece35029-bib-0086] Rocha, L. A. , Robertson, D. R. , Roman, J. , & Bowen, B. W. (2005). Ecological speciation in tropical reef fishes. Proceedings of the Royal Society of London B: Biological Sciences, 272(1563), 573–579. 10.1098/2004.3005 PMC1564072 15817431

[ece35029-bib-0088] Sbrocco, E. J. , & Barber, P. H. (2013). MARSPEC: Ocean climate layers for marine spatial ecology. Ecology, 94(4), 979–979. 10.1890/12-1358.1

[ece35029-bib-0089] Schultz, J. K. , Pyle, R. L. , DeMartini, E. , & Bowen, B. W. (2007). Genetic connectivity among color morphs and Pacific archipelagos for the flame angelfish, Centropyge loriculus. Marine Biology, 151(1), 167–175. 10.1007/s00227-006-0471-5

[ece35029-bib-0090] Smith, B. T. , Harvey, M. G. , Faircloth, B. C. , Glenn, T. C. , & Brumfield, R. T. (2013). Target capture and massively parallel sequencing of ultraconserved elements for comparative studies at shallow evolutionary time scales. Systematic Biology, 63(1), 83–95. 10.1093/sysbio/syt061 24021724

[ece35029-bib-0091] Spalding, M. D. , Fox, H. E. , Allen, G. R. , Davidson, N. , Ferdaña, Z. A. , Finlayson, M. , … Robertson, J. (2007). Marine ecoregions of the world: A bioregionalization of coastal and shelf areas. BioScience, 57(7), 573–583. 10.1641/B570707

[ece35029-bib-0092] Springer, V. G. (1959). Systematics and zoogeography of the clinid fishes of the subtribe Labrisomini Hubbs. Publ Inst Mar Sci Univ Texas, 5, 417–492.

[ece35029-bib-0093] Stamatakis, A. (2014). RAxML version 8: A tool for phylogenetic analysis and post‐analysis of large phylogenies. Bioinformatics, 30(9), 1312–1313. 10.1093/bioinformatics/btu033 24451623 PMC3998144

[ece35029-bib-0094] Tariel, J. , Longo, G. C. , & Bernardi, G. (2016). Tempo and mode of speciation in *Holacanthus *angelfishes based on RADseq markers. Molecular Phylogenetics and Evolution, 98, 84–88. 10.1016/j.ympev.2016.01.010 26876637

[ece35029-bib-0095] Tavera, J. J. , Acero, A. , Balart, E. F. , & Bernardi, G. (2012). Molecular phylogeny of grunts (Teleostei, Haemulidae), with an emphasis on the ecology, evolution, and speciation history of New World species. BMC Evolutionary Biology, 12(1), 57 10.1186/1471-2148-12-57 22537107 PMC3472276

[ece35029-bib-0096] Taylor, M. S. , & Hellberg, M. E. (2005). Marine radiations at small geographic scales: Speciation in neotropical reef gobies (*Elacatinus*). Evolution, 59(2), 374–385. 10.1554/04-590 15807422

[ece35029-bib-0097] Wainwright, P. C. , Santini, F. , Bellwood, D. R. , Robertson, D. R. , Rocha, L. A. , & Alfaro, M. E. (2018). Phylogenetics and geography of speciation in New World *Halichoeres* wrasses. Molecular Phylogenetics and Evolution, 121, 35–45. 10.1016/j.ympev.2017.12.028 29289544

[ece35029-bib-0099] Warren, D. L. , Glor, R. E. , & Turelli, M. (2010). ENMTools: A toolbox for comparative studies of environmental niche models. Ecography, 33(3), 607–611. 10.1111/j.1600-0587.2009.06142.x

