# Supplementary material for: Genomic, ecological, and morphological approaches to investigating species limits: A case study in modern taxonomy from Tropical Eastern Pacific surgeonfishes
Source: Ecol Evol. 2019 Mar 5;9(7):4001–12. doi: 10.1002/ece3.5029 (PMC6467843; doi:10.1002/ece3.5029)
Supplement: Supplementary file 1 [file ECE3-9-4001-s001.docx]

sssAppendix

**Figure S1.** Intermediate phenotypes found in this study. Faint spotting patterns can be found on various portions of the body, such as the head in LSUMNS 17879 (a), and the ventral portions of the body in LSUMNS 17801 (b). Both specimens are from Costa Rica.

**Figure S2.** Individuals from Coiba Island, Panama displaying differing amounts of spots while foraging. Red arrows highlight the irregular spotting pattern for the region. Photograph credit MAB.


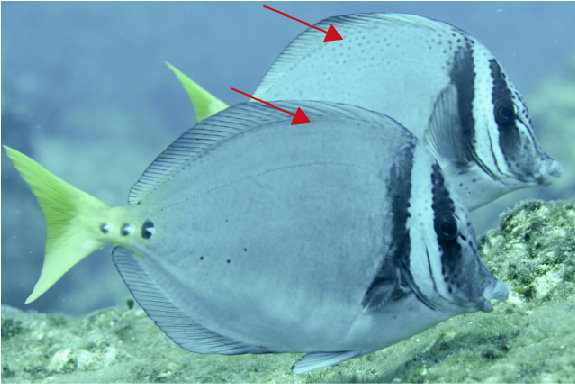


**Figure S3.** Maps showing the extent of the PC1 (A), and PC2 (B) layers used for abiotic niche comparisons, which span the entirety of the TEP and cover the realized region where these two surgeonfishes could disperse. Blue dots represent verified occurrence data for *P. punctatus*, and red represents occurrence points for *P. laticlavius*.

**Table S1.** Materials examined for morphological comparisons. Institution codes are as follows: CAS - California Academy of Sciences; CMN – Canadian Museum of Nature; LACM – Natural History Museum of Los Angeles County; LSUMZ – Louisiana State University Museum of Natural Science; MNHN – Muséum national d’Histoire naturelle; SIO – Scripps Institution of Oceanography. ^1^ Some specimens in these lots exhibited intermediate phenotypes. ^2^ Type material. ^3^ Only photographs examined to determine spotting pattern. ^4^ Specimens miss-identified, or lot contains a mixture of both phenotypes.

Taxon Institution Catalog Number N Country State/Province Locality Name

*P. punctatus*

CAS C86W51-58 3

CAS 1513^1^ 1 Mexico Colima Isla Socorro (Revillagigedo Islands)

CAS 2911 4 Mexico Sinaloa Mazatlan

CAS 16813 2 Mexico Baja California Sur Cabo San Lucas

CAS 19018 2 Mexico Baja California Sur North of Cabo San Lucas

CAS 79587^2^ 1 Mexico Baja California Sur Cabo San Lucas

CMN 1968 – 0720^3^ 1 Mexico Colima Isla Socorro (Revillagigedo Islands)

LACM 1231 5 Mexico Baja California Sur Buena Vista

LACM 1487 1 Mexico Baja California Sur Southwest of El Tule

LACM 1609 2 Mexico Baja California Sur Southwest of El Tule

LACM 26273 3 Mexico Baja California Sur Punta Pescadero

LACM 50636.003 1 Mexico Baja California Sur Punta Pescadero

LACM 50644.005 1 Mexico Baja California Sur Punta Pescadero

LACM 50667.044 1 Mexico Baja California Sur Bahia los muertos

LACM 8862.018 9 Mexico Baja California Sur Isla Ceralvo

LACM 9044.011 12 Mexico Guerrero Southeast of Papanoa

LACM 9321.001 1 Mexico Sinaloa Isla Los Lobos

LACM 9349.001 1 Mexico Sinaloa Isla Venado?

LACM 9361.001 1 Mexico Colima Manzanillo

LACM 31768.024 4 Mexico Baja California Pta. Entrada?

LSUMZ 17801^1^ 1 Costa Rica Guanacaste Isla Leoncillo

LSUMZ 17878^1^ 4 Costa Rica Guanacaste Playa Hermosa

SIO 05 – 56 1 Mexico Baja California Sur Isla Carmen

SIO 07 – 188 1 Mexico Baja California Sur Todos Santos

SIO 50 – 295 1 Mexico Baja California Sur Ensenada de los muertos

SIO 59 – 225 1 Mexico Baja California Sur Punta Pescadero

SIO 61 – 243 2 Mexico Baja California Sur Punta Los Frailes

SIO 62 – 8 13 Mexico Nayarit Isla San Juanito

SIO 62 – 9 2 Mexico Nayarit Isla San Juanito

SIO 62 – 19 1 Mexico Nayarit Isla María Madre

SIO 62 – 25 2 Mexico Nayarit Isla María Magdalena

SIO 62 – 55 3 Mexico Nayarit Isla Cleopha

SIO 62 – 56 1 Mexico Nayarit Isla Cleopha

SIO 62 – 66 1 Mexico Nayarit Isla Isabel

SIO 64 – 58 4 Mexico Baja California Sur Punta Tosco

SIO 70 – 155 1 Mexico Guerrero South of Zihuatenejo

SIO 75 – 529 8 Mexico Baja Sur California Bahia Los Frailes

*Prionurus laticlavius*

CAS 23762 1 Ecuador Galápagos Isla Santa Cruz

CAS 39287 1 Ecuador Galápagos Isla Pinzón

CAS 50087 2 Ecuador Galápagos Isla Fernandina

CAS 106369 1 Mexico? Colima Isla San Benedicto (Revillagigedo Islands)

CMN 1968 – 0718.12^1,3,4^ 2 Mexico Colima Isla Socorro (Revillagigedo Islands)

CMN 1968 – 899.21^3^ 3 Mexico Colima Isla Clarión (Revillagigedo Islands)

CMN 1968 – 0999.4^3^ 2 Mexico Colima Isla Clarión (Revillagigedo Islands)

CMN 1968 – 1008.13^3^ 1 Mexico Colima Isla Socorro (Revillagigedo Islands)

LACM 9351.002 4 Mexico Colima Isla Clarión (Revillagigedo Islands)

LACM 9358.001^4^ 1 Ecuador Galápagos Isla Santiago

LACM 9382.001 1 Ecuador Galápagos Locality not listed

LACM 31768.024^4^ 1 Mexico Baja California Pta. Entrada

LACM 31778.047^4^ 2 Mexico Colima Isla San Benedicto (Revillagigedo Islands)

LACM 31783.027^4^ 3

LACM 32096.009 1 Mexico Colima Isla Clarión (Revillagigedo Islands)

LACM 32097.023 1 Mexico Colima Isla Clarión (Revillagigedo Islands)

LACM 32494.029 2 Costa Rica Guanacaste Isla Cocinero (Murcielago Islands)

LACM 43865.001 1 Ecuador Galápagos Isla Española

LACM 45582.012 1 Ecuador Galápagos Isla Santa Cruz

LACM 45587.003 1 Ecuador Galápagos Isla Baltra

LSUMZ 17800 5 Costa Rica Guanacaste Isla Leoncillo

LSUMZ 17860^1^ 4 Costa Rica Guanacaste Playa Ocotal

LSUMZ 17879^1^  6 Costa Rica Guanacaste Playa Hermosa

LSUMZ 18090^1^ 1 Panamá Provincia de Panamá Isla Pacheca (Las Perlas Islands)

LSUMZ 18091 1 Panamá Provincia de Panamá Isla Pacheca (Las Perlas Islands)

LSUMZ 18094 2 Panamá Provincia de Panamá Isla Pacheca (Las Perlas Islands)

LSUMZ 18095^1^ 7 Panamá Provincia de Panamá Isla Pacheca (Las Perlas Islands)

LSUMZ 18096 1 Panamá Provincia de Panamá Isla Pachequilla (Las Perlas Islands)

MNHN A-7493^2,3^ 1 Ecuador Galápagos Isla Santa Fé

SIO 61 – 239 1 Mexico Baja California Sur Punta los Frailes

SIO 50 – 292 1 Ecuador Galápagos Isla Santa Cruz

SIO 53 – 148 1 Ecuador Galápagos Isla Fernandina

SIO 64 – 1017 1 Ecuador Galápagos Isla Santa Cruz

SIO 48 – 130 1 Panamá Provincia de Panamá Locality not listed

SIO 07 – 188 1 Mexico Baja California Sur Todos Santos

**Table S2.** Summary of STRUCTURE analyses for *K* = 1–5 ordered in decreasing likelihood values.

*K* Mean LnP(*K*) Stdev LnP(*K*) Ln’(*K*) |Ln’’(*K*)| Delta *K*

2 -14774.22 1.303 69.4 1380.04 1059.37752

1 -14843.62 0.766 – – –

5 -14922.76 133.425 206.38 – –

4 -15129.14 771.405 955.72 749.34 0.971

3 -16084.86 3075.458 -1310.64 2266.36 0.737

*COI Amplification*

All samples were sequenced for the mtDNA COI region following procedures outlined in Ludt et al. (2012). Briefly, COI segments were amplified with primers BOL-F1 (59 TCA ACY AAT CAY AAA GAT ATY GGC AC 39) and BOL-R1 (59 ACT TCY GGG TGR CCR AAR AAT CA 39) (Ward et al. 2005). Each 25ml reaction was comprised of approximately 10 ng DNA, 3.5 mM MgCl2, 1X buffer, 0.18 mM of each primer, 2.5 mM DNTP, and 2 units of GoTaq DNA Polymerase (Promega). Polymerase chain reactions were conducted using a temperature profile of a one minute denaturing step at 95ºC, followed by a 30 second annealing temperature of 45–52ºC depending on the species, and completed with an extension of 45 seconds at 72ºC, for 32 cycles. Samples were then purified and sequenced using both the forward and reverse primers.
